# Nature-Based Biomaterials and Their Application in Biomedicine

**DOI:** 10.3390/polym13193321

**Published:** 2021-09-28

**Authors:** Eoin Troy, Maura A. Tilbury, Anne Marie Power, J. Gerard Wall

**Affiliations:** 1 Microbiology, College of Science and Engineering, National University of Ireland, NUI Galway, H91 TK33 Galway, Ireland; eointroysuncroft@gmail.com (E.T.); mauratilbury@gmail.com (M.A.T.); 2 SFI Centre for Medical Devices (CÚRAM), NUI Galway, H91 TK33 Galway, Ireland; 3 Zoology, School of Natural Sciences, NUI Galway, H91 TK33 Galway, Ireland; annemarie.power@nuigalway.ie

**Keywords:** biomaterial, scaffold, tissue engineering, drug delivery, collagen, gelatine, silk, cellulose, chitosan, alginate

## Abstract

Natural polymers, based on proteins or polysaccharides, have attracted increasing interest in recent years due to their broad potential uses in biomedicine. The chemical stability, structural versatility, biocompatibility and high availability of these materials lend them to diverse applications in areas such as tissue engineering, drug delivery and wound healing. Biomaterials purified from animal or plant sources have also been engineered to improve their structural properties or promote interactions with surrounding cells and tissues for improved in vivo performance, leading to novel applications as implantable devices, in controlled drug release and as surface coatings. This review describes biomaterials derived from and inspired by natural proteins and polysaccharides and highlights their promise across diverse biomedical fields. We outline current therapeutic applications of these nature-based materials and consider expected future developments in identifying and utilising innovative biomaterials in new biomedical applications.

## 1. Introduction

Traditional biomaterials used in biomedicine, such as gelatine, silk and collagen, were derived from natural sources [1], with their first clinical applications dating to the 1950s [2]. While they have had enormous impact on patient quality of life to date, they are being continuously modified, exploiting advances in the fields of molecular and cellular biology and polymer chemistry, to improve their material properties, bioactivities and suitability for therapeutic applications [3]. Furthermore, as biomaterials expand into new applications such as drug delivery, tissue engineering, scaffolds and bioprinting [4], new and modified materials are being developed that can remain in intimate and productive contact with tissues in the body for long periods [5].

Biologically inert materials were originally favoured for biomedical applications on the basis of safety and stability. Years of clinical use have identified that even inert mate-rials may elicit damaging cellular and immunological responses, however [6,7]. As a re-sult, biomaterials must now, at a minimum, interact with their surrounding tissues, while in more advanced applications, they may be designed to interact with surrounding cells and tissues to promote tissue healing and regeneration. Biomaterials that are biologically inert and passive are augmented with drugs, growth factors or gene delivery vectors to manipulate cellular responses in vivo for greater therapeutic effect [8,9].

This review describes the broad range of current biomaterials that are derived from, or inspired by, natural proteins and polysaccharides. We summarise the diverse biomedical and biotechnological roles successes of these materials to date, in fields such as regenerative medicine and therapeutics. Finally, we consider potential future directions for the development and modification of novel biomaterials with broader and more effective biomedical applications.

### 1.1. Which Biomaterial?

Choosing a suitable biomaterial for, e.g., scaffold construction is critical for its success. In vivo target sites at which biomaterials are used differ greatly and include soft (ligament, skin, cartilage, muscle, nerve, tendon, vascular sites) [10] and hard (bone and teeth) [11] tissues with very different biological and physicochemical properties [12]. Traditional alloys, metals and ceramics with limited functionality are increasingly being replaced by more versatile materials, while polymeric biomaterials are replacing permanent prosthetics due to concerns about the lower biocompatibility of the latter and the need for revision surgeries [13]. Increasing numbers of polymeric biomaterials are emerging from synthetic and natural sources, with diverse applications in tissue engineering and regeneration, as well as in specialist fields such as drug delivery, nanotechnology and gene therapy [14].

Biodegradable polymeric biomaterials can be divided into natural and synthetic materials, based on their origin and whether they contain naturally occurring extracellular matrix (ECM) [15]. Commonly used synthetic polymers, such as polyglycolide (PGA), polylactic acid (PLA) and poly(lactic-co-glycolic acid) (PLGA), are often cheaper to produce at scale and have more homogeneous structures, mechanical strengths and degradation rates [16]. They lack natural binding sites for cells, however, resulting in lower bioactivities and increased in vivo rejection rates than natural biomaterials [17]. Natural biomaterials are sub-classified based on their composition and include protein-based materials such as collagen, gelatine, silk and fibrin, and polysaccharide-based materials such as cellulose, chitosan and alginate [5,15], as described in detail below.

### 1.2. Biomaterials in Biomedicine

Scaffolds that act as templates for tissue regeneration and guide the development of new tissues can be produced from a diverse range of materials (Table 1) [18]. Natural ECM can act as a porous 3D microenvironment “scaffold”, with its multitude of growth factors, effector molecules, enzymes and cellular adhesion motifs influencing cell proliferation, gene expression and intracellular signalling [19,20,21]. Due to the complexity and variability of ECM, however, scaffolds used in tissue engineering are typically more tailored to particular functions, such as promoting cell adhesion or differentiation [22,23]. Regardless of the clinical objective or environment, all biomaterial scaffolds share the following essential properties.

#### 1.2.1. Biocompatibility

Scaffolds should allow or promote cells to adhere, proliferate and spread before creating a new matrix, and must not elicit an inflammatory response that could lead to infection, longer healing times or patient discomfort [18].

#### 1.2.2. Biodegradability

Scaffolds should ideally be temporary templates which will be replaced by newly regenerated tissue [24]. Therefore, they should be biodegradable, resulting in non-toxic breakdown products which are safely excreted without interfering with normal bodily functions [18].

#### 1.2.3. Structure

Scaffolds must be highly porous to promote cell migration, waste dispersal, scaffold-tissue interaction and nutrient and fluid permeability [25]. Cell-binding ligands may be naturally present in ECM-derived scaffolds or incorporated into synthetic materials [18]. Pores must be large enough to allow infiltration of cells but small enough to establish a suitable cell density attached to the scaffold [18,26].

#### 1.2.4. Mechanical Properties

Scaffolds must have sufficient mechanical strength to maintain their structural integrity—including during transport, surgical handling and implantation [27,28,29]. Engineered scaffolds should typically mimic the mechanical properties of their target tissue [27] and therefore vary greatly between, e.g., soft tissue applications and bone or cartilage scaffolds [30,31].

#### 1.2.5. Manufacturing Technology

Scaffolds must be cost-effective to produce and easily scalable from laboratory production. Production must also be compatible with good manufacturing practice (GMP) standards [32] for translation from the laboratory to the clinic [33].

## 2. Protein-Based Biomaterials

In nature, an array of proteins play vital structural roles in living organisms, which has led to their incorporation in recent years into protein/polypeptide-based biomaterials based on their structural chemistry, cellular interactions or cell communication properties [101]. Non-structural proteins are also gaining attention due to their ability to modulate the functional properties of biomaterials. In this section, we review protein-based materials derived from natural sources and consider their increasing impact in biomedicine.

### 2.1. Collagen

Collagen is the most abundant structural protein in humans and animals. It makes up approximately 30% of all mammalian proteins and is an essential component of the ECM [102]. By virtue of its characteristic fibrillar structure, it provides structural support to hard and soft tissues, including cartilage, tendon, bone, ligament and blood vessels [103].

The collagen family consists of 29 distinct collagen types which are divided into four classes based on their composition and structural properties [102]. All types exhibit a characteristic triple helix structure, consisting of three α-chains comprised of more than 1000 amino acids and a repeating Gly-X-Y sequence. The glycine residues allow tight inter-molecular packaging of the α-chains while the X and Y positions are typically filled by proline and 4-hydroxyproline, respectively [104]. Of the 29 types, only types I, II, III, V and XI are known to form collagen fibres [103] and these are favoured in collagen-based biomaterials [105].

#### 2.1.1. Biological Characteristics

Collagen is weakly immunogenic, with fibrillose collagen exhibiting lower immunogenicity than smaller molecules due to the burial of potentially antigenic sites during its auto-polymerisation [103]. Removal of non-helical regions and cross-linking of collagen chains can be carried out to further reduce antibody recognition [106]. Cross-linking also provides stability and increases resistance to collagenase activity—properties which contribute to its biocompatibility and degradability in biomedical applications [103,107].

#### 2.1.2. Cell Binding

Specific peptide sequences in collagen bind four different types of cell-surface receptor. GPO (Gly-X-Y) motifs, where X and Y can be any amino acid residue but are most commonly proline [108], bind type 1 receptors, which includes glycoprotein VI [109]. GFO (Gly-Phe-Hyp) inter-act with type 2 receptors, which consist of collagen-binding integrin proteins and discoidin domain 1 and 2 [110,111]. Small molecule-binding cryptic domains in collagen [112] bind various integrins in type 3 receptors, while type 4 cell receptors bind non-collagenous domains within the protein [113]. Indirect cell-collagen interactions also promote cellular adhesion to the ECM, often via fibronectin (an ECM glycoprotein). Fibronectin was the first molecule on which the integrin-binding sequence RGD (Arg-Gly-Asp) was identified, which has since been found in many protein types, explaining their ability to bind collagen [103,114]. Receptor-binding motifs make an important contribution to the success of collagen as a biomaterial scaffold by aiding in cell seeding, adhesion and promoting cell differentiation and migration.

#### 2.1.3. Obtaining Collagen

Collagen can be obtained for biomedical applications from mammalian sources such as cows, pigs, rats and sheep [105,115], as well as human peripheral nerve tissue [116] or human placenta [117]. It is purified by decellularisation, in which cellular antigens from the natural collagen matrix are removed while maintaining the ECM shape and structure, or by an extraction, purification and polymerisation approach which yields more refined scaffolds [103]. Decellularisation can be achieved by physical, chemical and enzymatic processes [118]. Physical decellularisation disrupts cell membranes and promotes cell lysis by the use of rapid freezing or high pressure approaches, and may be combined with chemical steps to increase tissue penetration [118,119]. Chemical decellularisation involves the addition of alkalines, acids, detergents and chelating agents to remove the cellular components of ECM while enzymatic decellularisation utilises the proteolytic enzyme trypsin, nuclease enzymes and ethylenediaminetetraacetic acid (EDTA) to remove proteins and DNA and RNA [103,118]. As none of the methods yields an ECM entirely free of cellular material, several techniques are typically combined to produce a pure, decellularised ECM [103] suitable for use as ligament prostheses or cardiac valves.

Extraction and purification of collagen from natural sources can also be carried out by solubilisation and purification. Due to its covalent cross-linking, collagen exhibits low solubility in organic solvents and is typically solubilised in acidic (0.5 M acetic acid), neutral salt (0.15–0.20 M NaCl) or proteolytic solutions.

#### 2.1.4. Cross-Linking Collagen

Unlike decellularised collagen which has been naturally polymerised in vivo, extracted collagen must be cross-linked to increase its mechanical strength and resistance to enzymatic degradation. There are several well-established methods of cross-linking, including physical processes such as ultra-violet (UV) or thermal treatment [120], via chemicals such as formaldehyde and glutaraldehyde [121], and by the use of cross-linking enzymes such as transglutaminase [122]. The addition of biomolecules such as elastin [123], chitosan [124] and glycosaminoglycans (GAG) [125,126] during cross-linking can be used to improve the cell differentiation, migration, or proliferation characteristics, or mechanical properties of the resultant scaffolds [103,107].

#### 2.1.5. Sterilisation of Collagen

Whether purified via decellularisation or solubilisation, extraction and cross-linking, collagen must be sterilised for in vivo use. Due to its relatively fragile, temperature-sensitive structure, sterilisation methods can alter its molecular properties. Even low-dose gamma irradiation reduces its enzymatic resistance and mechanical strength, though the addition of glucose can mitigate these effects by mediating cross-linking [127]. β- and electron-beam irradiation, while less damaging than gamma irradiation, have still been reported to cause structural degradation, and reduced mechanical strength and resistance to enzymatic degradation [103]. Immersion of collagen in low concentrations of sterilant is becoming a popular sterilisation approach, with low concentration pancreatic acid effective for decellularised collagen, and an ethanol/antibiotic mix for extracted, cross-linked collagen. As no approach can completely avoid altering the collagen structure, however, it is essential to thoroughly investigate the effect of each method on the properties of the resultant material relative to its intended application [103].

#### 2.1.6. Recombinant Production

As collagen is primarily obtained from animal sources, the transmission of infectious disease is a concern in therapeutic applications. The recent COVID-19 pandemic exemplifies the dangers of zoonotic infections, while cases of bovine spongiform encephalopathy have resulted from the use of prion-contaminated bovine scaffolds [128]. Meanwhile, religious constraints surround the use of porcine and bovine materials, and up to 2%–4% of the world’s population may be allergic to porcine and bovine-derived collagen [129]. These factors, as well as the heterogeneity of natural collagen preparations, have led to the development of approaches to produce recombinant human collagen (rhCOL) [130,131].

*Escherichia coli* is the best-established expression system for recombinant proteins due to its ease of genetic manipulation, rapid growth rate, track record in protein production and suitability to scale-up [132]. It can produce a protein similar to human collagen which contains the characteristic Gly-X-Y sequences but differs from the native collagen through its lack of proline and lysine hydroxylation [133]. The failure of *E. coli* to carry out these post-translational modifications (PTMs) results in a collagen with reduced thermostability and limited fibre assembly, thereby restricting its usefulness in tissue engineering [131]. Cloning of prolyl and lysyl hydroxylase genes from the aquatic giant Mimiviridae virus family into *E. coli* has enabled the successful production of molecules with hydroxyproline and hydroxylysine patterns that are characteristic of human collagen and capable of supporting the growth of human endothelial cells [134], but despite this success, eukaryotic systems continue to dominate recombinant production of collagen.

Since the expression of human interferon in *Saccharomyces cerevisiae* in the 1980s [135], yeasts have been very successfully used in recombinant protein production. *S. cerevisiae* and *Pichia pastoris* are the two most commonly used yeast hosts [136] and their utility is due to their eukaryotic protein folding mechanisms and ability to carry out PTMs required for many proteins’ functioning. Like prokaryotic systems, they lack native lysyl and propyl hydroxylases [132] but co-expression of human hydroxylases enables them to produce rhCOL closer in structure and properties to native human collagen that that expressed in *E. coli* [132]. rhCOL from both *S. cerevisiae* and *P. pastoris* has been used to produce hydrogels for wound healing applications [131].

A variety of mammalian systems have also been investigated for the accurate production of human collagen. Chinese hamster ovary (CHO) cell-derived rhCOL was shown to reverse the disease phenotype of dystrophic epidermolysis bullosa (characterised by collagen deficiency within the skin) when administered intravenously, without eliciting an immune response in mice models of the disease [137]. Human HeLa cells [138] and embryonic kidney cells [139] have also been used to produce rhCOL types I, V and VII identical to native human collagen produced in vivo. Yields are much lower than from other expression systems, however, so non-human animal platforms are typically preferred. rhCOL has also been produced in transgenic animals, with mouse embryos transfected with a *COL1A1* gene found to secrete correctly folded rhCOL type I through their mammary glands [140]. Transgenic animal production systems are considerably more specialised and expensive than cell-based systems, however [132].

#### 2.1.7. Collagen-Based Scaffolds

Upon obtaining collagen, scaffolds of pure collagen, a collagen/natural polymer blend or a collagen/synthetic polymer blend can be produced [141].

Collagen types I, II and III have been electrospun into fibres at submicron to nanometre scale that replicate the biological properties of ECM. Collagen fibres with a 67-nm binding pattern, similar to native collagen, have been formed from electrospinning collagen type I [142], leading to the production of biomimetic scaffolds with tuneable porosity, mechanical strength and fibre alignment to topographically guide tissue formation. Electrospun collagen scaffolds have also been demonstrated to support cellular growth [142,143,144]. Pure collagen scaffolds have weak structural stability and mechanical strength, however. While this can be improved by cross-linking fibres using UV irradiation or dehydrothermal treatment (high temperature exposure under a vacuum), care must be taken to ensure no residues of toxic cross-linking chemicals such as glutaraldehyde remain in the scaffold [141].

Scaffolds can also be constructed by blending natural or synthetic polymers with collagen for improved mechanical properties. Natural polymers already established in tissue engineering include fibroin, chitosan and silk. Chitosan is a non-immunogenic, biodegradable, positively charged polymer which has been combined with collagen to form scaffolds with excellent mechanical and biological properties, as well as excellent compatibility with a range of seeded cell types [145,146,147,148]. Synthetic polymers utilised in blended collagen scaffolds include PLA and polyethylene glycol (PEG) [141]. In this scenario, the synthetic polymer typically improves the mechanical properties and structure of the scaffold while collagen provides cell signalling and binding sites crucial for tissue repair.

##### Collagen Sponges

Collagen sponges are produced from insoluble collagens extracted from cows or pigs. The scaffold is created by freeze-thawing alkali and aqueous acid collagen solutions containing up to 5% dry matter, with the rate and temperature of freezing determining the pore size and structure: rapid freezing at extremely low temperatures cause cracking to occur in the collagen, resulting in small channels and a highly fibrous structure, while slower freezing and higher temperatures causes the collagen to have large, non-uniform pores and more continuous channels [103].

Sponges are ideal for use in wound care as they adhere smoothly to the wound bed, are capable of absorbing large volumes of exudate, maintain a moist environment and shield against physical trauma and bacterial infection [104]. Cross-linking with glutaraldehyde or other polymers can be used to increase their mechanical strength. As collagen promotes invasion of immune/inflammatory cells such as neutrophils, sponges have potential uses in treating burns, diabetic ulcers and at donor sites [39]. Loading of sponges with exogenous growth factors can also be used to improve wound healing, such as platelet-derived growth factor (PDGF) and fibroblast growth factor (FGF) to promote capillary formation and epidermal wound healing, respectively [40,41]. Collagen sponges have also been used to provide sustained delivery of antibiotics such as vancomycin [42] and gentamycin [43] to treat sepsis, and intra-vaginal delivery of retinoic acid to avoid systemic effects in the treatment of cervical dysplasia [44].

##### Collagen Hydrogels

A hydrogel is a 3D network of polymers which can hold significant volumes of fluids. The main therapeutically-relevant properties of polymeric hydrogels are their water retention, due to the hydrophilic functional groups on their polymeric backbone, their resistance to dissolution due to their cross-linking and their similar flexibility to natural ECM [149]. Due to their structural similarity to tissue, collagen hydrogels are frequently investigated as biomimetic 3D scaffolds to support cell growth [150].

The amphoteric (adsorbs to both anions and cations) nature of collagen type I fibres enables them to form hydrophobic, dipole-dipole, electrostatic and hydrogen interactions which lead to gel formation in aqueous systems [150]. The gels can typically be dissociated by collagenases or changes in temperature or pH [151]. While the natural cross-linking of collagen confers proteolytic resistance and mechanical strength, additional physical or chemical cross-linking can be necessary to prevent enzymatic degradation [152]. Of these approaches, glutaraldehyde cross-linking via lysine and hydroxy-lysine residues [153], sometimes used in combination with 1-ethyl-3-(3-dimethylaminopropyl) carbodiimide (EDC) cross-linking of carboxyl and amine groups [150], yields the most stable hydrogels and can be designed to vary the mechanical properties and function of the hydrogel [150], though some cytotoxicity has been reported [154].

**Figure 1:**
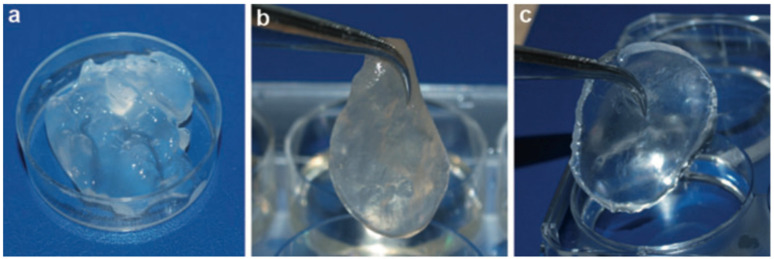
Collagen hydrogels: (**a**) non-cross-linked, (**b**) glutaraldehyde cross-linked and (**c**) EDC/NHS cross-linked [150].

Figure 1 illustrates non-cross-linked collagen compared with glutaraldehyde cross-linked and EDC cross-linked collagens.

Collagen hydrogels are attractive scaffolds in tissue engineering due to their retention of cells and bioactive molecules. They have found widespread application in cartilage and bone tissue engineering, such as acting as carriers for bovine chondrocytes [34] to provide structural support and pain-free articulation of cartilage [35]. As chondrocytes produce cartilage ECM, their transplantation in collagen hydrogels can be used to treat a variety of articular cartilage defects [36] or to engineer bone tissue [37]. Osteoblasts derived from calf metacarpus periosteum, have also been demonstrated to proliferate and migrate within a 3D collagen hydrogel without any loss of viability over three weeks, as well forming a bone-like ECM containing osteonectin, osteocalcin and new collagen type I [37]. Collagen type I hydrogel-mediated treatment of bone defects in rat dorsal nasal bones was demonstrated to lead to the growth of a thin layer of bone after six weeks [155]. Blending of synthetic polymers such as polyvinyl alcohol (PVA) and polyacrylic acid (PAC) with collagen in hydrogels can be used to improve the mechanical strength of the natural polymers and the biocompatibility of the synthetic molecules [1]. In one such example, PVA was blended with collagen and used to form sponges, films and hydrogels, which were loaded with growth hormone. Release of the hormone was monitored in vitro by enzyme-linked immunosorbent assay and could be tuned by altering the collagen content of the gels [156].

**Figure 2:**
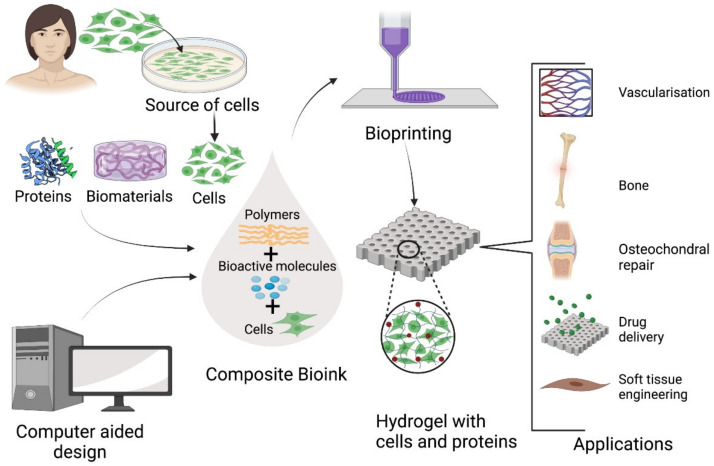
Bioprinting of natural polymers, frequently in combination with cells and/or biomolecules to fine-tune or increase in vivo activity, has potential to provide carefully designed, highly structured materials for tissue and organ engineering applications.

Collagen type I is commonly used as in bioprinting but its slow gelation rate at physiological temperatures means that it must typically be used in combination with biomolecules that improve its structural integrity [4] (Figure 2). Collagen-alginate bioinks have been demonstrated to have increased mechanical strength and to accelerate the proliferation of human chondrocytes for articular cartilage repair, [38] while printed collagen-alginate hydrogels have also demonstrated sustained release of antibacterial drugs [45].

##### Collagen Films/Membranes

Collagen films of 0.01–0.5 mm thickness can be produced by drying bovine collagen that has had its telopeptides (nonhelical regions that flank collagen’s triple helix) removed, followed by a series of enzymatic and chemical cross-linking steps [39,157]. Their main applications are as barriers to protect wounds or ulcers, while simultaneously releasing therapeutic drugs. Drugs can be loaded onto films via covalent or hydrogen bonding or through simple entrapment and the films can be easily sterilised without affecting their mechanical properties [39]. Collagen films are well established in wound dressing applications, as well as reinforcing compromised tissues and guiding tissue regeneration. They have also been used to deliver antibiotics [158,159] and, as collagen-coated polyurethane (PU) films, to promote attachment and proliferation of fibroblasts [160], which stimulates further collagen synthesis and the formation of new connective tissue and ECM. Individual films can also be easily combined into multiple-layered membranes which can release molecules such as PDGF at constant rates for up to 100 h in vivo to aid wound healing and tissue regeneration [107], or human growth factors to support healing of diabetic ulcers in murine models [161]. Collagen-based scaffolds have also been utilised as a resorbable template, alone or with additional components such as hyaluronic acid [162] or synthetic polymers [163,164], to regenerate the meniscal template of the knee, with promising in vivo outcomes [165].

Collagen-based materials are currently to the fore amongst biomaterials used in regenerative medicine and tissue engineering, based largely on their low immunogenicity, high biocompatibility and structural versatility. Advances in extraction and scaffold formulation have led to increasingly diverse applications of collagen in fields such as wound healing, drug delivery and tissue regeneration. Future research is likely to focus on improving the mechanical strength, drug delivery capabilities and biodegradability of collagen-based scaffolds in order to enhance their in vivo efficacies.

### 2.2. Gelatine

Gelatine is a well-characterised, biocompatible and biodegradable polymer which is formed by disintegration and denaturation of natural collagen, typically of bovine or porcine origin [166]. It is commonly utilised in food and cosmetic production as a cheaper alternative to collagen and has extensive pharmaceutical applications. It is a derivative of type I fibrillar collagen and contains up to 92% pure protein, as well as mineral salts and water [46]. It exhibits several advantages over its parent collagen in therapeutic applications, including reduced immunogenicity [167], increased solubility in aqueous systems and ease of transition from solution to gel at temperatures of 30 °C [168].

#### 2.2.1. Gelatine Structure

The structure of gelatine depends on the source and method of denaturation of the parent collagen [168]. This leads to heterogeneity of gelatine and batch-to-batch variability in its molecular weight from a thousand to greater than a million Dalton [166,169]. Denaturing collagen forms a gelatine solution with very low viscosity, which forms a gel at temperatures below 37 °C. This reversible process of thermo-responsive gelation results from peptide coils transitioning into helices due to the high numbers of Gly-Pro bonds within the structure [170]. As the gels return to a liquid state at temperatures above 37 °C, this makes natural gelatine unstable for in vivo use, though numerous cross-linking approaches have been developed to stabilise the macromolecular structure and avoid its rapid degradation in host tissues [169], as outlined below.

Mammalian gelatine is preferred for in vivo applications due to its high concentration of cell-binding domains, which create an excellent substrate for recruitment and attachment of adherent cells. Meanwhile, gelatine is less antigenic than collagen due to its lower composition of phenylalanine and absence of tyrosine and tryptophan which form aromatic rings and radicals that can promote an antigenic response [171].

#### 2.2.2. Obtaining Gelatine

Gelatine is produced by thermal denaturation and hydrolysis of collagen. Heating to only 40 °C disrupts the interior structure of newly formed or highly soluble collagen [169], whereas non-soluble collagen requires an additional hydrolysis treatment using acid or alkali solutions [169]. Soaking in dilute acidic solutions yields gelatine type A while submersion in alkali solutions produces gelatine type B, with the former more similar to collagen in isoelectric point and amino acid composition [172].

#### 2.2.3. Cross-Linking Gelatine

As with collagen, gelatine can be cross-linked using chemical, physical or enzymatic approaches. Physical cross-linking involves physical gelation via heat and pH changes as described above. To avoid the reversibility of physical gelation upon temperature or pH changes, chemical methods have been developed to produce a more stable polymer. Glutaraldehyde cross-linking is typically the preferred strategy as glutaraldehyde is easily accessible, cheap and rapidly boosts the mechanical strength of gelatine [151]. Chemical cross-linking can leave behind traces of potentially toxic chemical agents, however, leading to investigation of natural cross-linking agents such as caffeic acid, tannic acid, genipin and grape seed proanthocyanidin. Genipin, derived from the fruits of *Gardenia jasminoides* [173], is far less toxic than glutaraldehyde and achieves comparable mechanical properties in the cross-linked gelatine, but is limited by its high cost and formation of a dark blue pigment which can constrain its biomedical use [46,174]. Enzymatic approaches have been used to synthesise highly stable gelatine structures, with transglutaminases in particular favoured due to their abundance in nature and ability to produce a mechanically strong product [175].

#### 2.2.4. Gelatine Biomaterials

Gelatine is favoured in biomedical applications due to its commercial availability, low price, high solubility, biocompatibility, biodegradability, the presence of cell-binding domains and its lack of antigenicity or toxicity to cells [46]. Its disadvantages, however, surround its poor mechanical properties, lack of thermal stability, greater susceptibility to some proteases than collagen and faster degradation [176]. To overcome these disadvantages, advanced manufacturing techniques and cross-linking approaches are used to improve the thermal and mechanical stability, biocompatibility and overall bioactivity of native gelatine.

##### Gelatine Microparticles

The ability of gelatine to form a gel, its biocompatibility and its biodegradability make it ideal for the production of microparticles. Gelatine microparticles are extensively used as drug carriers due to their ease of production, stability and lack of toxicity, as well as their ability to interact with multiple bioactive compounds [177]. While smaller particles are used to protect and control the release of bioactive molecules in vivo [47], such as growth factors to stimulate cell proliferation and differentiation [46,48], larger microparticles, with modified surfaces for improved cell attachment and differentiation, can be used as “microcarriers” of cells, e.g., delivery of embryonic stem cells to aid bone regeneration [53].

**Figure 3:**
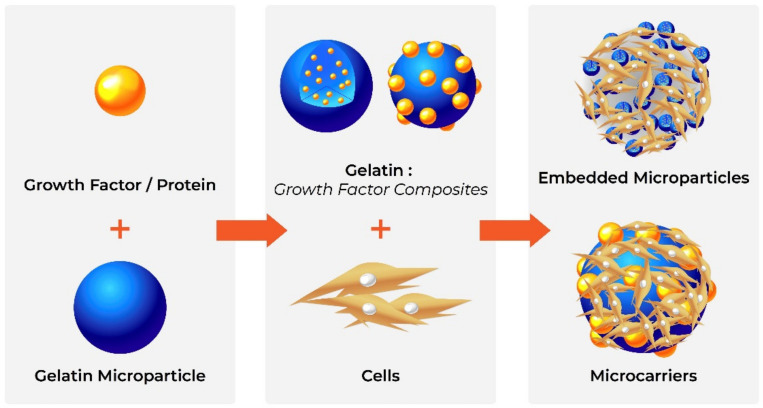
Loading of gelatine microparticles with growth factors and/or cells to direct cellular differentiation (based on [46]).

Gelatine microparticles are traditionally produced by techniques such as solvent evaporation, spray drying and precipitation, though these may cause denaturation [47] or leave solvent traces in the final product [178] (Figure 3). Therefore, improved production methods such as water-in-water emulsification have been developed [177].

##### Drug Delivery

The ability of gelatine microparticles to deliver anti-inflammatory [49], antibacterial [50] and antineoplastic [51,52] agents is well established. While encapsulation in the microparticle typically improves a drug’s pharmacokinetic profile and efficacy [179], a major challenge associated with nano- and micro-scale drug carriers is that they are commonly phagocytosed in vivo [51]. PEGylation has shown promise in protecting gelatine microparticles against opsonisation (particle attack from phagocytic immune response) and reducing their immunogenicity [51], as demonstrated in their improved delivery of doxorubicin and reduced cytotoxicity in a mouse model of pulmonary metastasis [180], and in sustaining release of ibuprofen in vivo, reducing the need for repeated injections [181].

Gelatine microparticles have also been used in optimised targeting of drug delivery, which is particularly important to avoid side effects and toxicity of oncology treatments. Magadala and Amiji [182] introduced an epidermal growth factor receptor (EGFR) recognition sequence into the gelatine amino acid backbone, leading to gene delivery to and uptake by EGFR-expressing Panc-1 human pancreatic adenocarcinoma cells, in an approach with clear potential to improve safety and efficacy of pancreatic cancer treatment [182].

Overall, gelatine is a versatile, biocompatible and widely available biomaterial which offers versatility across a range of tissue engineering and drug delivery applications. Given recent advances in targeted drug delivery, it is easy to envisage the continued development of gelatine-based systems in oncological applications.

### 2.3. Silk

Silk is a fibrous protein which has been used in the textile industry for centuries. It is naturally produced by arthropods, including silk “worms” which in fact are butterflies and moths (order Lepidoptera), and members of the class Arachnida (approx. 34,000 species of spiders). These organisms produce silk in specialised endothelial cells, followed by secretion into the lumen of their glands and spinning into fibres to build cocoons, nets, traps and webs [183]. The best-characterised silks are from the orb spiders *Nephila clavipes* and *Araneus diadematus* and the silkworm *Bombyx mori* [184]. The mechanical strength of spider silks is much greater than that of silkworm silks, but difficulties in cultivating predatory spiders has led to silkworm silk being more commonly used in the commercial silk industry, with spider silks (particularly their spidroin components) generally produced recombinantly [54]. Like collagen, silk is a fibrous protein and characterised by a repetitive primary sequence, which in turn leads to homogeneity in its secondary structure.

#### 2.3.1. Fibroins from *B. mori* Silk

**Figure 4:**
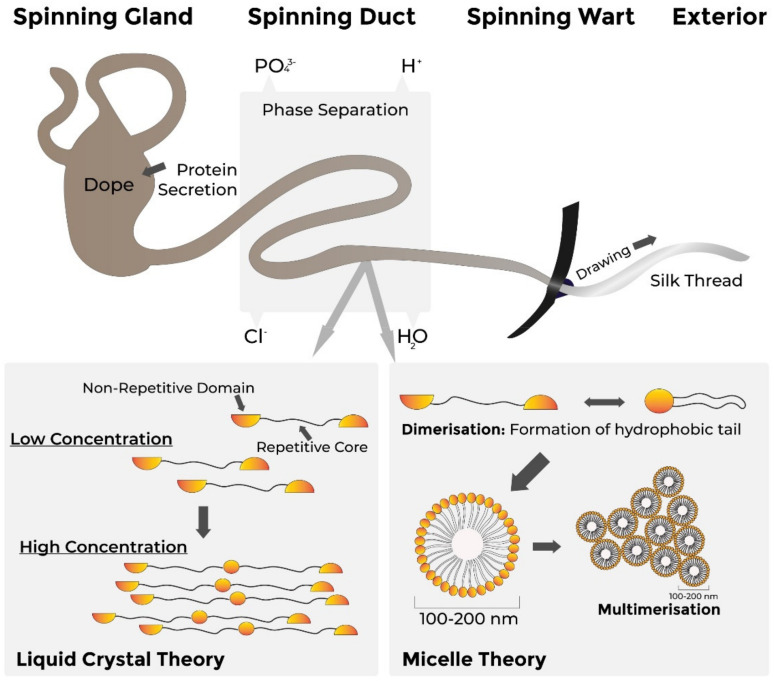
Silk spinning process (upper level) with liquid crystal and micelle theories of fibre formation (lower panels). A solution of silk protein is secreted into the spinning gland. During thread formation, the solution is passed through a channel in which ion exchange and phase separation occurs. Drawing of the thread causes fibre formation, which may be assembled by crystalline alignment of proteins (left) or micelle formation and assembly (right) (based on [189]).

Naturally produced *B. mori* silks are produced by larvae which grow for up to six weeks before they spin silk fibres to form cocoons which protect the larvae against predation, moisture and microorganisms (Figure 4) [185]. Harvesting and unravelling of the cocoons is then carried out to obtain the silk fibres, of which silk fibroin, the main component protein, is of particular interest in drug delivery, wound healing and tissue engineering applications [54].

Silks contain two or more fibroin proteins containing hydrophilic light (25 kDa) and hydrophobic heavy (325 kDa) chains linked by disulphide bonds [54]. The proteins are enclosed in a glue-like coat known as sericin which fuses the fibres to form the cocoon casing and must be removed by boiling in sodium carbonate [54]. The fibroins occur in a series of interlocked nanofibrils which combine to form microfibrils of 20–200 nm in diameter, with friction between the nanofibrils leading to the impressive mechanical strength of the silk fibres. Glycoprotein P25 is also non covalently attached and plays an essential structural role in the silk fibroin [186]. The hydrophilic light chains, dominated by alanine (14%), serine (10%), glycine (9%) and *N*-terminal acetylated serine residues [54], and the hydrophobic heavy chains, featuring glycine (46%), alanine (30%), serine (5.5%) and valine (2%), form a crystalline, “anti-parallel” β-sheet, fishnet-like structure, which is fundamental to the high mechanical strength of the silk nanofibre [187].

Once sericin has been removed, silk-based materials are usually produced for biomedical applications by the dissolution of the fibrils/threads in calcium nitrate, lithium thiocyanate and 4-methylmorpholine *N*-oxide, followed by dialysis against pure water to remove electrolytes. The final fibroin solution can be stored at low temperature (months) or room temperature (weeks) prior to the production of scaffolds or other biomaterials [54].

The nanofibrillar silk structure provides excellent strength, toughness, weight, flexibility and extensibility [188]. Its extremely high strength-to-density ratio is ideally suited to biomedical applications. Remarkably, silk fibres have been reported to be stronger than Kevlar and of equal strength to steel and nylon [189,190]. Silk fibroins are insoluble in water and most organic solvents, and very stable at high temperatures, with side-chain amino acid groups and peptide bonding reportedly stable up to 200 °C [191]. They carry no infection risk and, while some materials have failed to gain clinical approval due to immunogenicity, this may have been due to the outer sericin layer as the core silk materials invoke very mild inflammatory or immune responses while sericin can trigger allergic and immune reactions, and the release of tumour necrosis factor-alpha (TNF-α) [192]. Removal of sericin reduces the immunogenic response to silk fibroin to less than that of collagen or synthetic PLGA [54]. Silk fibroins exhibit a slow loss of mechanical strength in vivo due to enzymatic degradation, with non-toxic degradation products [54]. Overall, silk-based biomaterials can be completely biodegradable several weeks post-implantation and 100% bioresorbable following 11–12 months [193].

#### 2.3.2. Silk Fibroin Biomaterials

The properties of silks mean that they are outgrowing their traditional applications in textiles and expanding into proof of concept experiments and pre-clinical investigations in areas such as wound healing and gene therapy.

Silk films can be easily produced from fibroin stock solutions by spin-coating or layer-by-layer methods and have been utilised as scaffolds in numerous tissue engineering applications. Silk films functionalised through chemical binding of RGD domains promoted bone formation when scaffolds were seeded with osteoblasts [194], while silk and collagen films exhibited equivalent abilities to support cell binding, differentiation and physiological morphology in human cell culture [195,196]. A silk/chitosan composite film containing 40%–50% chitosan was demonstrated to be a promising matrix for use in wound dressing and artificial skin material [55], and seeding with human adipose-derived stem cells may promote repair of soft tissue wounds in murine models [22].

Electrospinning of silk fibres can generate fibres with diameters from sub-micron to nano-scale, with large surface areas capable of incorporating nano-sized molecules. Spinning is usually carried out using the initial silk fibroin solution and fibres are treated with methanol and washed with deionised water to form the characteristic β-sheet structures [54,197]. Electrospun silk fibroin fibres can be woven or compiled (non-woven) into biomedical scaffolds which are gaining interest in wound healing due to their oxygen permeability, fluid drainage and water retention [54]. By incorporating silver nanano-particles, a silk fibroin non-woven mat exhibited antimicrobial properties against skin commensals *Staphylococcus aureus* and *Pseudomonas aeruginosa* that were equivalent to commercially available wound dressings containing a 20x higher silver concentration [198]. Growth factors can also be loaded onto mats to stimulate epithelialisation. Loaded mats were shown to provide sustained release of epidermal growth factor (EGF) in a 3D model of human skin, leading to 98% wound closure after 48 h compared with 8% in non-treated control and 26% in silk only groups [199].

The analogous natural biosynthetic process (Figure 4) and shear thinning properties of silk fibroin also make it suited to extrusion bioprinting [4]. A silk–gelatin combination hydrogel, in which gelatin was provided bulk [200], was used to print a human ear which exhibited in vitro and in vivo biocompatibility and maintained its shape and volume for three months while promoting cellular infiltration and tissue integration [201].

#### 2.3.3. Spider Silks (Spidroins)

Spider silk is one of the toughest known biomaterials. It owes its mechanical properties to the organisation of the large spider silk proteins, one of which is spidroin [202]. While natural fibres typically display lower strength and stiffness than synthetic materials, spider silks are capable of absorbing up to three times more weight than Kevlar [203] —and have been utilised in biomedicine for centuries, with ancient Greeks and Romans using spider silk poultices to cover wounds and act as styptics (anti-antihaemorrhagics) [56]. They have been more challenging to commercialise than insect (*B. mori*) silks, however, as they are produced in much smaller quantities and spiders’ predatory nature makes them more difficult to grow in large numbers. There are numerous types of spider silks produced from separate glands on the body, with some spiders producing several forms for distinct prey capture, shelter and dragline functions, but the best characterised is the major ampullate gland (MA) silk produced by spiders in the *Nephila* genus, notably *Nephila clavipes*. Spider silk is also much more elastic than insect silk and can resist more mechanical stress caused by bending which, in addition to its shape memory, allows spider silk to outperform many natural and synthetic fibres in specific biomedical applications [189], as outlined below. The lack of sericin proteins in spider silk may also present some advantages.

##### Structure of Spider Silk (*N. clavipes*)

MA silk is one of six silk fibres produced by the genus *Nephila* and has a very defined nanostructure composed of the two main structural proteins, major ampullate spidroin-1 (MaSp1) and -2 (MaSp2) [203]. The primary structure consists mainly of proteins with large numbers of hydrophobic and non-polar amino acids [204] and highly repetitive amino acid sequences of up to 50 residues which make up 90% of the overall silk protein [189,205]. An alternating hydrophilic and hydrophobic pattern in the core domain dominates the primary structure and is thought to be necessary for phase separation during the silk spinning process [189]. There are four oligopeptide motifs which are often repeated within MA silks: (i) (GA)n/(A)n; (ii) GPGGX/GPGQQ; (iii) GGX; and (iv) a “spacer” sequence of charged amino acids [206]. Structural and functional analysis of these sequences is ongoing but it is clear that (GA)n/(A)n sequences form α-helices in solution and β-sheet structures when assembled into fibres [189], while the GPGGX/GPGQQ and GGX sequences may encode amorphous rubber-like structures within the protein [207]. Intermolecular disulphide bonds stabilise protein dimers and trimers and may contribute to silk protein assembly [58].

When first secreted from glands, spider silks exhibit no stable secondary or tertiary structure [208]. Long repetitive sequences allow intra- and inter-molecular interactions with other proteins and enable the secondary, tertiary and quaternary structures to form through the silk spinning process. As the final silk thread structure contains high electron density areas within areas of noticeably lower densities [209], it is postulated that the former areas, corresponding to a high concentration of β-sheets, promote mechanical strength, while the latter, found in regions with amorphous structures, provide elasticity to the silk fibres [189,210].

##### Spider Silk Assembly

Within the glands, spider silks have a random coil structure which quickly assembles and becomes water-insoluble when secreted into the spinning duct [211]. Assembly involves folding of the protein and is based on aligning and packing of single silk proteins, with the alignment of repetitive hydrophobic core regions and multimerisation through shear forces and the proteins’ terminal domains.

A number of alternative mechanisms of silk fibre assembly have been proposed (Figure 4). The first indicates that silk proteins form micelles of up to 200 nm in diameter due to their amphiphilic (both hydrophilic and hydrophobic) domains [212]. Dimerisation of these proteins, with each dimer forming a hydrophobic tail, can prime self-organsiation into micelles, followed by the combination of micelles into larger-diameter globules which pass through the spinning duct to elongate and form fibres [189]. The second hypothesis suggests that fibre assembly is based on the crystalline alignment of proteins within the laminar flow of the spinning ducts, with high concentrations of monomeric and multimeric proteins passing through the spinning duct and alignment of these proteins causing liquid-crystalline behaviour in the spinning dope (polymer solution) [189], with fibres drawn from the spinning duct through solvent loss.

##### Obtaining MA Silk

Due to the predatory nature of spiders and their low silk yields in captivity, recombinant expression approaches have been developed to obtain larger quantities of spider silk. Initial expression attempts in *E. coli* [18] were typically limited by low yields, poor solubility of the translated products and inclusion body formation, translation inefficiencies due to differences in codon usage between the spider genes and the bacterial host, and depletion of tRNA pools [213].

As mammalian cells can typically express and process much larger proteins than prokaryotes, recombinant MaSp1 and MaSp2 silk genes were expressed in murine mammary glands [214]. While use of a goat signal sequence enabled production of MA silk in the milk of the transgenic mice, yields were too low for large-scale exploitation [214,215].

Silkworms have also been investigated as expression hosts for spider silk [216]. Using the well-established Baculovirus gene expression system, spider silk protein was successfully produced in *B. mori* larvae [217]. Yields were again limited by the poor solubility of the protein [217] though this could be overcome using a chimeric *N. clavipes* MaSp1 protein, resulting in expression and spinning of composite *B. mori* silk fibres with mechanical strength equal to native MA silk [218].

#### 2.3.4. Spider Silk Biomaterials

Spider silks have found application in suturing, in coating of implants and as foams and hydrogels due to their versatility, mechanical and environmental stability, and biocompatibility [56]. MA silk fibres have been used to suture severe tendon ruptures, which are notoriously slow to regenerate. Traditional suture materials are not compatible with interior suturing, can cause immune reactions and may lose mechanical stability over time [56]. Silk fibres from *N. clavipes* were braided and used to secure tendon injuries [57], with tensile strength equal to traditional sutures and no reduction of strength after more than 1000 rounds of fatigue testing. No immune response was detected, emphasising the potential of spider silk threads to replace many traditional suturing methods.

##### Organ Reconstruction

MA silk from *Nephila* species has been investigated in pre-clinical trials for use in bladder reconstruction [219]. Single silk threads were cross-woven to form a mesh which supported primary human urothelial cell (HUC) adhesion and growth in vitro without additional biological stimuli. No changes were detected in epithelial-to-mesenchymal transition or expression of fibrosis-associated genes while the HUCs could elongate on the material and form a bladder mucosal layer without any toxicity [219], demonstrating the potential of this silk type in bladder reconstruction.

##### Spider Silk Coatings

Coatings made from recombinant spider silks have also been deployed as “bioshields” to reduce the foreign body reaction associated with the use of silicone in medical and cosmetic procedures [59]. This formation of a capsule around the implanted material causes contraction of the implant, leading to distortion of surrounding tissues [220] and infection, pain and unsightly cosmetic results in up to 10%–26% of silicone implant recipients [221], particularly as biocompatibility of the implanted material decreases over time [59]. Coating of silicone implants with a micro-thin layer of recombinant *Araneus diadematus* spider silk protein eADF4(C16) [58] significantly increased their biocompatibility in the critical initial months post-implantation [59]. This lack of immunogenicity of the eADF4(C16) spider silk makes it an ideal candidate for improving the biocompatibility of implanted silicones and other implanted devices and prosthetics currently hindered by immunological reactions.

Silk-based biomaterials have proved themselves one of the most versatile and promising protein-based biomaterial categories for biomedical applications. They have exceptional biocompatibility, mechanical properties, biodegradability and cell-binding capabilities. Silks from both silkworm and spider sources have excelled across multiple applications in bone regeneration, tissue engineering/regeneration, suturing and improving the biocompatibility of implanted devices. Future research may focus on translating that success into medical devices and prostheses, which may lower incidences of rejection and infection, resulting in improved outcomes and postoperative patient comfort.

## 3. Polysaccharide-Based Biomaterials

Polysaccharide biomaterials can be obtained from a wide variety of sources and have found application in cosmetics, food industries, biomedicine and pharmaceuticals [222]. The biomaterials include alginates, the structural cell wall constituent of many seaweeds [223], chitin and its derivative chitosan from crustacean and fungal sources [224], and cellulose, the most abundant polysaccharide on earth [225]. Many polysaccharides exhibit biocompatibility, stability and biodegradability, as well as widespread natural abundance, which lends them potential in biomaterials applications [226,227].

### 3.1. Cellulose

Cellulose can be sourced from cotton, wood and other plant-based sources, as well as from some bacterial sources [228]. It is the most abundant biopolymer in nature, with almost 30 billion tonnes of natural cellulose biomass produced annually [229]. Plant cellulose (PC) exists as a primary material of the plant cell wall while bacterial cellulose (BC) is chemically identical to PC but secreted by bacterial from genera such as *Acetobacter*, *Pseudomonas* and *Sarcina* [225]. Both forms of cellulose have tuneable biochemical, mechanical and physical properties, as well as biocompatibility, good mechanics and bioactivity [230].

#### 3.1.1. Molecular Structure of Cellulose

Cellulose is an unbranched carbohydrate polymer in which β-D-glucopyranose residues are linked by β-1,4-glycosidic bonds [225]. Different crystalline forms can be distinguished by their overall structure: cellulose I is the native form of cellulose and is defined by inter- and intra-sheet hydrogen bonds and van der Waals interactions, parallel β-(1,4′)-D-glucopyranose chains and two main crystalline structures—triclinic cellulose Iα and monoclinic cellulose Iβ [230]. Cellulose II, meanwhile, is produced by alkali treatment of cellulose I and exhibits altered hydrogen bonding and antiparallel cellulose I subunits [231,232]. Cellulose III is produced by ammonia or amide treatment of cellulose I or II and defined by the hydrogen bonding between sheets [233]. The molecular structures of the cellulose classes greatly affect their physical and mechanical properties. Cellulose I is the strongest, with a Young’s modulus of 138 GPa, and cellulose III the weakest (58 GPa) [234], while stability also decreases from cellulose I through to cellulose III due to the different microfibrillar arrangements within the molecules [230].

#### 3.1.2. Plant and Bacterial Celluloses

While PC and BC are both cellulose I, their differences in structure and microfibre arrangement confer different properties on the molecules, including their purity, ability to retain water, mechanical strength, porosity and crystallinity [230]. BC is considered the “pure” cellulose as PC typically contains impurities in the form of residues from protective hemicellulose [235], while it also contains a higher proportion of cellulose Iβ, making it less crystalline [236]. BC microfibrils are noticeably smaller than those of PC, resulting in BC having a higher capacity for water retention and being much more porous than PC, though the properties of each material are influenced by their source and production methods [230].

#### 3.1.3. Nanocellulose

For biomedical applications, celluloses formed as nanofibre networks are preferred [235]. This increases the surface area of the celluloses and strengthens interactions with polymers and biomaterials [237]. Nanocelluloses are cellulose extracts composed of structural nanoscale materials which take advantage of the properties of both cellulose and nanomaterials [238]. While the health and environmental risks of nanoparticles are well established [239,240], nanocellulose fibres are irreversibly fixed within the molecular structure of cellulose. Nanocelluloses have been reported to exhibit very low toxicity to date but toxicology studies are continuing, and inhalation of cellulose nanocrystals (CNC) has been reported to cause pulmonary inflammation in animals due to self-aggregation of nanocellulose and its slow degradation [238].

There are three different types of nanocellulose described to date: CNC and cellulose nanofibrils (CNF), both of which are produced by the breakdown of PC using refinery techniques and shear forces, and bacterial nanocellulose (BNC) which is produced by bacteria [235,238].

##### Obtaining Nanocellulose

CNC and CNF are produced from plant sources such as wood, hemp, cotton, algae and tunicin. Chemically induced destructuring such as by acid hydrolysis removes the amorphous cellulose regions while preserving their highly crystalline structure of CNCs [238]. For CNF, production involves mechanical destructuring of the cellulose structure by grinding or homogenisation, followed by chemical and enzymatic treatments [238], which can completely remove cellulose nanofibrils from microfibres within the cellulose structure. Unlike CNCs, CNFs retain both amorphous and crystalline cellulose regions and so appear as longer—up to 10–100 nm, depending on the source, mechanical defibrillation process and chemical treatment—and more flexible chains than CNCs [241].

While CNCs and CNFs are both produced via destructive processes, BNC is synthesised by bacterial species in a form which requires very little processing [242]. Chains of glucose are produced within the bacteria and secreted through pores in the cell envelope. The glucose chains combine to form microfibrils which aggregate further to create BNC with typical diameters of 20–100 nm [242].

##### Properties of Nanocellulose

Nanocellulose is characterised by its ordered crystalline and disordered amorphous regions, with cellulose chains within the crystalline regions providing stiffness, and chains within the disordered amorphous regions adding flexibility to the molecule. CNC is stiffer and more rigid than CNF or BNC, with a Young’s modulus of 100–200 GPa, which compares favourably to that of steel (200–220 GPa) [243]. CNF and BNC, meanwhile, exhibit lower Young’s moduli of approximately 100 GPa due to their amorphous regions [238]. Nevertheless, these values indicate potential for application of all three nanocellulose types as load-bearing biomaterials.

During formation of nanocrystals, cellulose chains orient in a unidirectional, parallel manner within the fibrils, resulting in a robust hydroxyl functionality at one end and a reducing moiety at the other. Meanwhile, glucose units display multiple active hydroxyl groups, which are responsible for hydrogen bonding between glucose chains and can be up to 10 times more reactive than regular OH groups [244]—and can be functionalised to add surface properties to nanocellulose [245].

There have been very few biocompatibility studies on cellulose nanofibrils. Some studies report only evaluations of material biocompatibility in terms of cultivation, growth and activity of cells, such as with CNC hydrogels [238], while others have focused on haemocompatibility: 2,2,6,6-tetramethylpiperidine-1-oxyl (TEMPO)-oxidised CNCs could regulate variables of blood metabolites while achieving acceptable haemocompatibility [246]. Subcutaneous injection of BNC in rats, including with infiltrated fibroblasts, identified no detectable immune foreign body response after 12 weeks [247]. Cellulose degrades slowly within the body, however, due to the absence of cellulolytic enzymes [238]. Oxidised cellulose is more vulnerable to hydrolysis, leading to attempts to enhance BNC degradation by oxidation, which proved promising in in vitro analyses [248].

#### 3.1.4. Nanocellulose as a Biomaterial

**Figure 5:**
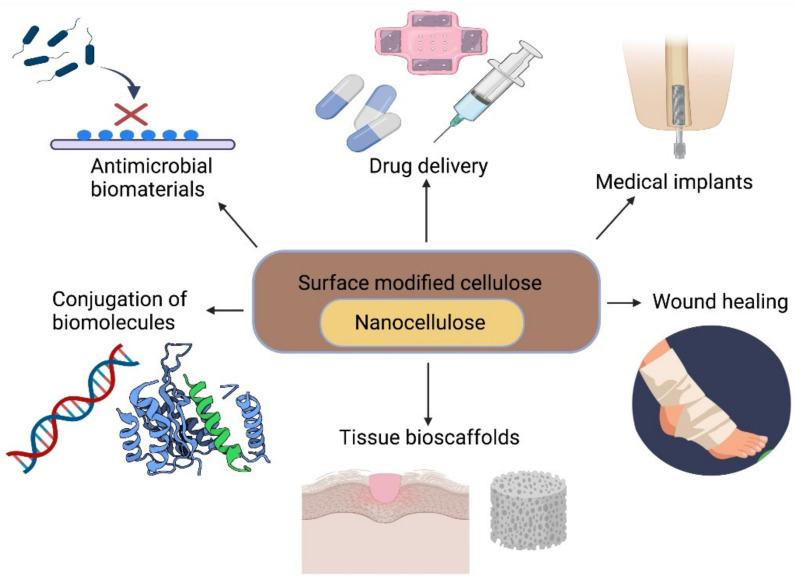
Examples of the uses of cellulose as a biomaterial (based on [249]).

In recent years, significant progress has been made in the use of nanocellulose in lesion repair, as scaffolds to support cell culture and in tissue repair/regeneration (Figure 5), with a predicted annual market value of approximately $97 billion [238].

##### Nanocellulose in Lesion Repair

Bodin et al. [60] compared the mechanical properties of a BNC gel with collagen implants commonly used to treat degenerative meniscal lesions. The BC gel, grown to a thickness of 5–15 mm, exhibited much greater load-bearing ability than the collagen, as well as an ability to be moulded into a meniscus shape and promote cell migration [60]. An ear-shaped BNC prototype was also produced using a reverse ear mould, demonstrating that BC could successfully mimic the mechanical properties of native ear cartilage and could be moulded to produce ear replacements in a patient-specific manner [61].

##### Nanocellulose as Artificial Blood Vessels

The production of artificial blood vessels is an area of interest (with BNC in particular) due to the mechanical strength and blood biocompatibility of nanocellulose-based materials [238]. Zang et al. [62] produced a BNC artificial blood vessel of 100 mm length and 1 mm thickness using *Gluconacetobacter xylinum*. Extensive physical and structural analysis of the tubular scaffolds indicated that they displayed satisfactory thermal stability, an extensive nanofibre architecture and an adaptability to in vivo environments suited to use as blood vessel replacements [62]. CNC or CNF cannot be fabricated directly into tubular scaffolds and usually require the use of a matrix. CNC/fibrin composites have been reported to form small diameter, tubular scaffolds, with the fibrin matrix adding strength in vitro [63]. Tubular scaffolds of 0.7–1.0 mm wall thickness have also been fabricated from PU/CNF biocomposites, with CNF used to reduce the thrombogenicity and increase elasticity of PU [64]. The scaffolds were successfully utilised as prosthetic blood vessels in the carotid artery of an endocrine neoplasia patient [64]. Given their success in this instance, it is surprising that PU/CNF tubular scaffolds have not been investigated further. Nevertheless, nanocellulose has clear potential application in creating artificial blood vessels but large-scale in vivo testing is required to address critical, still unanswered questions about their long term stability, potential postoperative complications and thrombogenicity [238].

##### Nanocellulose as a Drug Excipient and Drug Delivery

Celluloses have been used as excipients for condensing drug-loaded matrices into pharmaceutical tablets for oral administration for many years. Despite this, nanocelluloses remain a relatively new biomaterial in the field. Pure CNC prepared by acid hydrolysis has been shown to bind large quantities of water-soluble tetracycline and doxorubicin, followed by sustained release over 24 h [65]. By modifying the crystalline regions of CNC via binding of cetyltrimethylammonium bromide (CTAB) to give the nanocellulose a neutral charge, CNC could also be used to bind and release, over 48 h, the hydrophobic anticancer drugs paclitaxel, docetaxel and etoposide. CTAB-modified CNC could also bind and be taken up by KU-7 (urothelial carcinoma) cells, indicating their potential use in targeted drug delivery [65]. CNF and BNC were al-so found to be better suited to spray drying of tablets than commercially available micro-crystalline cellulose, with much lower porosity [66] and improved flexibility, foldability and mechanical properties [67], respectively, than traditional tablet coatings.

##### Nanocellulose Wound Dressings

BNC, in particular, has attracted a lot of interest in wound care as it appears to stimulate the proliferation of a number of human cell types, including human adipose-derived stem cells, while exhibiting minimal cytotoxicity. Tissue regeneration and capillary formation were accelerated in wound beds treated with BNC-based biomaterials compared to commercial wound dressings (Figure 5) [68,69]. BNC wound dressing materials have been also demonstrated to promote accumulation of ECM in rat ulceration models, leading to contraction of the wound and improved healing [70], and to adhere extremely well to burn sites, limit “dead space” and create an ideal healing environment [71]. Given their promise in promoting wound healing, nanocelluloses have also been combined with materials such as cotton gauze [72], gelatine [73] and collagen [74] to create biocomposites with enhanced healing capabilities.

Unlike most applications of nanocelluloses, BNC-based biomaterials have moved past clinical trials and into common clinical practice. Their primary use is in the treatment of non-healing lower extremity ulcerations, where the use of BNC biomaterials has been reported to reduce healing times from a mean of 240–390 days to 80 days [238]. Many BNC-based wound healing products are currently available on the market with some, such as BioFil (burn and ulcer therapy) and Gengiflex (periodontal disease treatment), having dual functions in hydration and absorption to maintain an ideal healing environment [238]. Nanocellulose wound dressings with antimicrobial properties have also been developed by binding antimicrobial agents to nanocellulose by physical or chemical approaches. Silver and lysozyme are the most commonly used antimicrobials, with the former capable of being incorporated into nanocellulose by simple chemical reduction and impregnation techniques to yield materials which display strong antimicrobial activity while maintaining the biocompatibility necessary for practical applications [75,76,77].

CMC and cellulose nanocrystals have also been incorporated into numerous bioinks to improve their viscosity for printing and to provide mechanical strength to printed hydrogels [250]. This has enabled materials suited to wound dressing applications to be printed [4], as well as more anatomically complex structures such as a human ear and sheep meniscus, in a study in which alginate was incorporated for cross-linking, and human chondrocytes bioprinted in the ink exhibited 86% viability after 7 days of 3D culture [251].

Cellulose and nanocellulose clearly have widespread applications across wound healing, drug delivery and as substitute implants, due largely to their biocompatibility, versatility and tuneable chemical and physical characteristics. Their inherent lack of degradability when used as scaffolds may be the main limitation of nanocellulose biomaterials, so that their greatest promise may reside in drug delivery and wound healing, especially when combined with antimicrobial agents for improved effectiveness.

### 3.2. Chitosan

**Figure 6:**
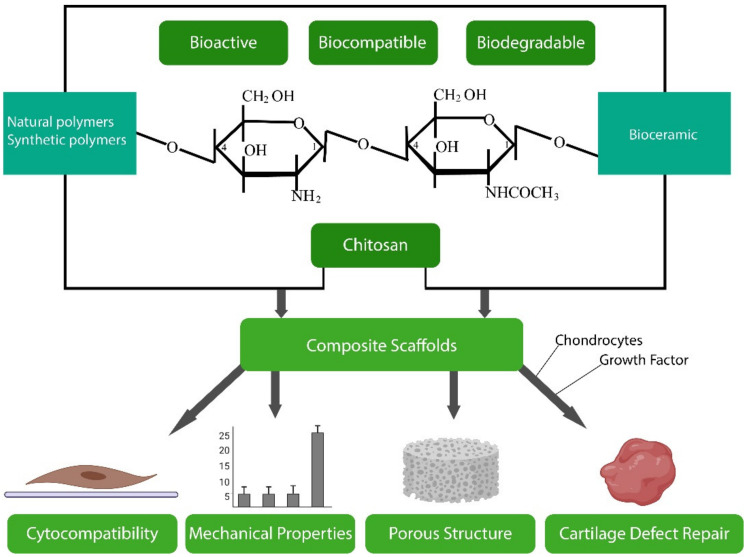
Composition of the chitosan polymer and examples of properties that influence its biomedical uses (based on [261]).

Chitosan is a unique biopolymer, derived from chitin, the second most abundant natural polymer on earth, which has gained significant attention in recent years for its potential in tissue engineering applications [252]. Both chitin and chitosan are found in the exoskeleton of crustaceans such as crabs and in the cell envelope of plants such as fungal hyphae (mushrooms) [250]. Chitin is a semi-crystalline linear polysaccharide copolymer composed of repetitive β-(1-4)-2-acetamido-2-deoxy-D-glucopyranose units in which the amines are completely acyetlated. Chitosan is a derivative of chitin with repeating β-(1-4)-2-amino-2-deoxy-D-glucopyranose units in which the *N*-acetyl glucosamine residues of chitin are completely deacetylated, giving rise to *N*-glucosamine [250]. Chitosan usually occurs as a copolymer of (1-4)-2-acetamido-2-deoxy-β-D-glucan (*N*-acetyl D-glucosamine) and (1-4)-2-amino-2-deoxy-β-D-glucan (D-glucosamine) units (Figure 6) [253]. To be classified as chitosan, chitin must have a deacetylation degree (DDA) of at least 60%, i.e., should contain at least 60% D-glucosamine residues [254]. Although discovered in the 1800s, chitin has been much less developed in biomedicine than structurally similar cellulose, which is largely attributable to its rigid structure and insolubility [250]. Chitosan is soluble in mild acidic solutions, however, and this, together with its amenability to modification, biodegradability and biocompatibility, has led to its extensive application in ophthalmology, wound healing/dressing and tissue engineering [250].

#### 3.2.1. Obtaining Chitosan

Chitosan is produced by deacetylation of its parent molecule chitin. While typically carried out using concentrated sodium hydroxide and sodium borohydride to prevent reactive species generation and chitin depolymerisation [250], numerous modifications have been described which alter the DDA of the resultant molecule [255], leading to changes in its physical and chemical properties [256,257]. Its chemical properties can then be further modified by adding functionalities to the abundant hydroxyl and amine groups on the chitosan backbone, such as addition of lactobionic acid to cause chitosan to become fully water-soluble [258], or oligopeptide addition [259] or carboxylation [260] to develop functionalised chitosans for tissue engineering applications.

#### 3.2.2. Biological Properties

Though a polysaccharide, chitosan exhibits multiple amino groups which render it degradable by human proteases such as lysozyme in vivo [262]. In addition, eight human chitinases have been described to date, of which three can degrade chitosan in vivo [263]. The byproducts of degradation are non-toxic oligosaccharides of varied sizes which are excreted via normal metabolic processes [264].

Chitosan is well known for its antimicrobial and antifungal properties, which make it attractive for use in biomedical scaffolds. The basis for its antimicrobial activity is uncertain but its positive charge is believed to interact with the negatively charged cell surfaces of microorganisms and inhibit both uptake and excretion of materials [265]. Low molecular weight chitosan is also reported to penetrate bacterial cell walls and bind to DNA via its protonated amino groups, leading to disruption of essential microbial activities such as RNA synthesis [266].

Recent in vitro and in vivo studies have highlighted the antitumor activity of chitosan, which is thought to result from an increase in interleukin (IL)-1 and IL-2 secretion due to the infiltration of mature cytotoxic T lymphocytes [267,268]. Chitosan has also been shown to be involved in inducing apoptosis and thus influencing the direct killing of tumour cells: it prevented adhesion and proliferation of primary melanoma A375 cells and caused apoptosis of metastatic melanoma RPMI7951 cells through inhibiting caspase activity and upregulating apoptosis regulatory genes such as Bax [269]. Expression of CD95 receptors was also upregulated on RPMI7951 cells following chitosan administration, which made the cells highly vulnerable to FasL-induced apoptosis [269].

Chitosan can aid blood clotting and reduce pain via blocking of nerve endings [270]. These haemostatic properties are attributed to negatively charged red blood cells being attracted to the protonated amine groups, leading to blood cell aggregation and clot formation, which quickly halts bleeding [270,271] Aside from stimulating red blood cell coagulation, studies have shown that chitosan can enhance platelet activation and aggregation [271] via a mechanism which has not been elucidated but is dependent on chitosan’s positive charge [272]. Chitosan is also well known for its mucoadhesive properties, and its ability to bind many surfaces within the body [273].

#### 3.2.3. Chitosan Biomaterials

The biodegradability and antimicrobial, anti-tumour, haemostatic and mucoadhesive properties of chitosan have led to it finding numerous applications in biomedicine and pharmaceuticals, as outlined below.

##### Chitosan in Wound Healing

Chitosan is used in wound healing due to its versatility and the fact that it can be readily manufactured both into 2D films and fibres and 3D scaffolds such as hydrogels and sponges [274]. As well as its haemostatic capabilities, chitosan induces neutrophil migration [275] and stimulates proliferation of dermal fibroblasts to accelerate re-epithelialisation in wound healing applications [276]. Antimicrobial chitosan hydrogels developed as a coating for vascular prosthetic grafts have been demonstrated to provide resistance to *E. coli* in vitro and in vivo [79,80]. Meanwhile electrospun chitosan/poly (vinyl alcohol) composite nanofibre wound dressings were found to stimulate fibroblast attachment and proliferation with a complete absence of cellular toxicity [78].

##### Chitosan-Based Molecule Delivery

The exceptional mucoadhesive properties of chitosan also lend the material to drug delivery applications. Nasal administration of chitosan microspheres containing peptides has been shown to promote transport of the encapsulated peptides through the nasal barrier, leading to enhanced adhesion and absorption on the nasal epithelium [81]. Delivery of diphtheria toxoid in chitosan microparticles in this manner significantly improved systemic and local immune responses to the toxoid in mice models through enhanced absorption arising from chitosan’s mucoadhesive properties [81]. Chitosan is also increasingly used to deliver micronutrient polyphenols which exhibit pharmacological potential as anti-inflammatory agents in Alzheimer’s disease, cardiovascular disease and some forms of cancer [82]. Encapsulation of tea-derived polyphenols in chitosan nanoparticles for oral administration protected the polyphenols against degradation and oxidation within the gastrointestinal tract while enhancing their uptake by endothelial cells [83]. Chitosan nanoparticles have similarly been shown to protect peptide drugs such as insulin and enhance their stability and controlled release [84].

Chitosan’s excellent biodegradability and antimicrobial, anti-tumour, haemostatic and mucoadhesive properties enable it to create suitable environments to promote wound healing. It has also been shown to have potential in the delivery of drugs and vaccines. While chitosan nanoparticles look particularly promising, additional studies are required on their toxicity to humans in particular.

### 3.3. Alginate

Alginates are anionic and hydrophilic polymers which are amongst the most widely available biosynthesised materials on earth [85]. They occur as cell wall constituents of brown algae such as *Laminaria hyperborea* and *Macrocystis pyrifera*, and *Pseudomonas* and *Azotobacter* bacterial species [277]. They are linear biopolymers consisting of homogenous (poly-G, poly-M), heterogeneous (MG) or block-like arrangements of 1,4-linked β-D mannuronic acid (M) and 1,4 α-L-guluronic acid (G) residues [277]. Their widespread availability, low cost, minimal toxicity, biodegradability and profound gelating ability have led to them being approved by the U.S. FDA for use as a biopolymer in regenerative medicine and tissue engineering [278].

#### 3.3.1. Structure and Properties

**Figure 7:**
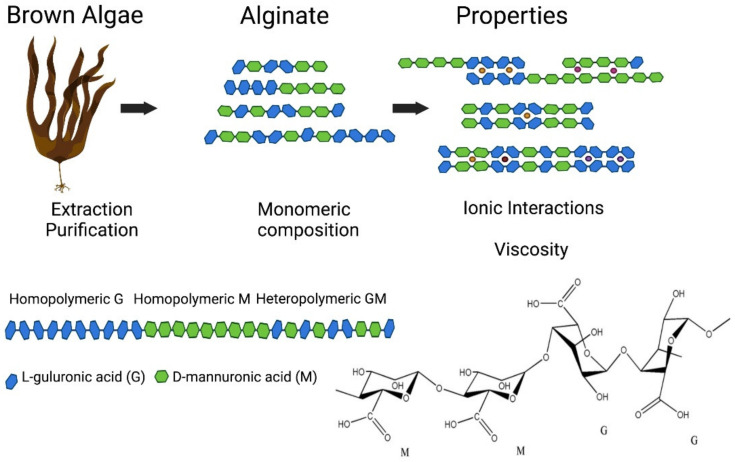
Alginate is extracted and purified from a wide variety of brown algae. It is composed of α-L-guluronic acid (G) and β-D-mannuronic acid (M) blocks, the precise combination of which impact the properties of the alginate material and its potential biomedical utility. Image is based on [286].

Alginates linear copolymers consist of blocks of residues of both (1,4)-linked β-D-mannuronate (M) and α-L-guluronate (G) which can occur as continuous G or M residues, i.e., (GGGGGG) or (MMMMMM), or as alternating residual patterns, i.e., (GMGMGM) (Figure 7) [85]. There are currently over 200 unique alginates produced for different purposes with varying G and M contents and overall block lengths [223].

G/M ratios, length of G blocks and the sequence pattern of GM block repeats all affect the physical properties of alginate hydrogels [279]. Alginates from different sources produce highly variable gels [85], with many bacterial alginates, such as those extracted from *Azotobacter* species, extremely rigid due to very high concentrations of G-blocks [280], which in turn impacts their potential application in drug release and cell encapsulation.

Despite extensive research on the biocompatibility of alginates, the effect of the molecular structure on biocompatibility remains uncertain. While alginates high in M blocks have been reported to be highly immunogenic and to stimulate cytokine production at 10-fold higher levels than alginates with a high G block content [281], other in vivo studies have detected no immunogenic or inflammatory reaction to implanted alginates [282,283,284]. It is likely that inflammatory reactions may be due to impurities within the alginate preparation rather than the material itself, with potential impurities including endotoxins, polyphenolic compounds, non-algal proteins, arsenic and heavy metals [285].

Alginate is not degraded by mammals as they lack the alginase enzyme responsible for cleaving the polymer chains [85]. Additionally, mammals lack receptor sites for alginate polymers, rendering alginate gels inert [287], though hydrogels with increased biological activity can be produced, as outlined below.

#### 3.3.2. Obtaining Alginate

Most commercially available alginates are derived from the above kelp species, typically harvested from natural populations as cultivation is considered too expensive for industrial production. While alginate biopolymers can make up almost 40% of the dry weight of the brown seaweeds [288], their quantity and quality vary with species, season and age of the seaweed. Alginate is typically extracted in aqueous alkali [85], filtered, precipitated and recovered as a water-soluble sodium alginate powder [224]. While modification of the natural structure to improve its physical and chemical properties is gaining attention [289,290], this review focuses on natural alginates from brown algae given their current dominance in the market.

#### 3.3.3. Alginate Hydrogels

While other biomaterials are utilised in many different formats, such as sponges, films and microparticles, alginate is primarily used in the form of hydrogels. These hydrogels can be formed by a variety of gelling mechanisms to modify their functionality.

##### Ionic Cross-Linking Gelling Strategies

Alginate hydrogels are most commonly prepared by the addition of ionic cross-linking agents such as divalent Ca^2+^ cations, typically in the form of calcium chloride [85]. The cations form highly coordinated bonds with the G blocks of the alginate chains, leading to alignment of the G blocks of adjacent chains into a cross-linked “egg-box” formation which provides the gel structure [291]. Buffers rich in phosphate groups, or lowering the reaction temperature, are often utilised to slow the reaction, which enhances the ordering of cross-links and increases the mechanical stability of the hydrogel [291,292]. As noted above, the origin and structure of the alginate influences cross-linking, with alginates with higher G-block contents undergoing more extensive cross-linking and yielding more rigid gels [293].

Stability issues may arise when gels formed through ionic cross-linking are incubated under physiological conditions for extended periods of time, however, due to exchange reactions with surrounding monovalent cations [85]. Ca^2+^ ions released via this process can promote haemostasis, while the resultant poorly cross-linked gel may also lead to aggregation of red blood cells and platelets [294]. Therefore, some focus has been turned to cross-linking gels through covalent means for increased structural stability.

##### Covalent Cross-Linking

Cross-linking of alginates using PEG amines of different molecular weights has been investigated to produce gels with a broader range of mechanical properties. Increasing the cross-linking density/amount (weight) of PEG led to improved mechanical strength and increased Young’s moduli in gels [85,295]. Controlling the cross-linking density and using multiple cross-linking agents can regulate the overall mechanical properties and swelling of gels [295]. As an example, poly(aldehyde guluronate) (PAG) gels cross-linked using multifunctional agents, such as poly(acrylamide-co-hydrazide) (PAH), displayed significantly enhanced mechanical properties and lower degradation than similar gels cross-linked using the bi-functional cross-linker adipic acid dihydrazide (AAD), apparently due to greater binding of PAH to the PAG gel [296].

##### Enhancing Biodegradation Properties

As mammals lack the enzymatic capacity to break down alginate chains, this jeopardises the potential biomedical usefulness of alginates, as biodegradation and safe excretion from the body are primary concerns for biomaterials in vivo [287]. While ionically-linked alginate gels dissolve over time due to the release of divalent ions into the surrounding medium, many commercial alginate gels have molecular weights larger than the threshold for renal clearance, which disrupts their excretion from the body [85,297].

Multiple approaches have been developed to produce biodegradable alginates for in vivo applications. Partial oxidation of alginate leads to increased degradability in aqueous environments [287] due to changing the conformation of the alginate backbone to an open-chain adduct without disrupting its gelling characteristics. In another approach, alginates can also be constructed entirely from G-blocks isolated from whole alginates, resulting in a degradable gel [298] better suited to biomaterial production.

##### Enhancing Cellular Interaction

As mammals lack cell receptors for alginate polymers, coupling of molecules such as fibronectin [299] and collagen [300] may be necessary to promote adhesion [287]. While coupling of whole molecules has proved difficult, attaching short amino acid chains which promote ECM adhesion has been more successful. RGD-modified gels promote increased proliferation of myoblasts [301], with RGD concentration and M/G ratio of the gels affecting cell behaviour and cell phenotype development [302]. Altering ligand densities can modify biological effects, with endothelial cell differentiation enhanced in gels containing intermediate concentrations of ligands [303] and hepatocyte differentiation stimulated only at much lower ligand densities [304]. Gel stiffness and rigidity also influence ligand clustering and cell adhesion [287], proliferation and differentiation [305] and morphology [306].

#### 3.3.4. Alginate-Based Biomaterials

Alginate gels have been widely applied across various fields of biomedicine, including wound healing, tissue/bone regeneration and as model systems to study mammalian cell culture [85].

##### Alginates as Wound Dressings

Ionically cross-linked alginate gels can be processed by freeze-drying to produce foam-like porous sheets and fibrous dressings which provide a moist environment more suited to wound healing than traditional dressings [86]. The dressings begin dry but quickly absorb excaudate fluid from the wound bed to begin a “re-gelling” transition and form a gel which provides excellent healing conditions and supports formation of granulation tissue and re-epithelialisation. Commercial alginate wound dressings include Algisite™ (Smith and Nephew) and Comfeel Plus™ (Coloplast) [85], the success of which has led to the development of alginate-based dressings designed to release bioactive compounds. Incorporation of a regulator of human keratinocyte proliferation, dibutyryl cAMP (DBcAMP), to oxidised alginate gels allowed for its sustained release into the surrounding medium, leading to full wound healing and complete re-epithelialisation over 10 days in rat models, compared to 15 days with control alginate gels [87]. Similarly, incorporation of stromal cell-derived factor-1 (SDF-1) to alginate dressings accelerated healing in porcine wound models—and with reduced scar formation [88]. Incorporation of silver into alginate-based dressings improved antimicrobial activity and inhibited infection, as well as enhancing binding of matrix metallo-proteases-2 (MMP-2) and pro-inflammatory cytokines TNF-α and IL-8 [89].

##### Alginates in Drug Delivery

Alginate gels have been used for sustained delivery of both low molecular weight chemical and protein drugs. The former diffuse rapidly from nanoporous (pore size ~5 nm) [90] alginate gels, e.g., anti-inflammatory drug flurbiprofen is readily released from partially oxidised and ionically cross-linked alginate gels but its release is significantly delayed from ionic and covalently cross-linked alginate [91]. While the chemical structure of the drug predominantly dictates its release kinetics, some drugs, such as a number of anthracenediones used in chemotherapy, can interact with alginate or its cross-linkers and only be released upon breakdown and complete dissociation of the gel; others, however, such as methotrexate, form no interactions with alginate and are released almost immediately via diffusion [52,85]. In an innovative approach that holds promise for transporting and safely releasing a broad array of hydrophobic drugs, composite alginate hydrogels were produced by encapsulating oil-in-water microemulsions in hydrophilic gels [93], enabling the hydrophobic oil core to solubilise the hydrophobic drug ketoprofen and the Ca^2+^ cross-linked outer gel matrix to act as a delivery vehicle.

Alginate has also proven an excellent delivery vehicle for protein-based drugs as proteins are easily incorporated into alginate gels without denaturation and the gels protect the proteins from degradation and allow their controlled release. Protein release rates are typically fast due to the gels’ hydrophilic properties and porous structures [85], though some growth factors—in particular molecules such as vascular endothelial growth factor (VEGF) and FGF which bind heparin—interact with the gels, leading to a more sustained release [94,95]. Much current research is dedicated to controlling the release of proteins from gel vehicles, particularly molecules which do not have heparin-binding properties.

The fast release of most protein drugs from alginate gels can be slowed by gel cross-linking techniques which can increase the incidence and strength of protein-gel interactions [85]. Insulin, for example, is completely released from alginate gel micro-spheres outside the gastrointestinal environment due to dissociation of the carrier vehicle at pH 6.8 [307]. By developing alginate/polyanionic polymer (cellulose acetate phthalate) blends followed by chitosan coating, however, insulin is protected in gastric pH 1.2 conditions and can be successfully delivered to the intestine. Similarly, layering *B. mori* silk fibroin over alginate microspheres has been utilised to achieve a mechanically rigid structure which protects enclosed proteins and prevents their diffusion [308].

##### Alginates as Protein and Cell Carriers for Tissue Engineering

Alginate gels have been widely utilised to transport proteins and cells for regeneration or engineering of tissues and organs in the body. As molecules smaller than 5 nm are quickly released from the porous alginate gel whereas those larger than the pore size are only released following gel dissociation, plasmid DNA molecules of approximately 100 nm in diameter are released from alginate gels only upon degradation [309].

Alginate gels have been used to stimulate angiogenesis and partly relieve ischemia in murine tissue through sustained and localised release of growth factors such as VEGF [94]. Gels have also been used to effect sequential delivery of growth factors for angiogenesis, with VEGF released prior to PDGF to stimulate early angiogenesis and blood vessel formation, followed by their enhanced formation and functionality in ischemic mice models [96].

Alginate gels are also attractive in bone regeneration as they can fill irregular shapes that other scaffolds may not and can be introduced into the body non-invasively and easily modified to carry cell-adhesion ligands or growth factors. Their inability to bear weight is of some concern [287] but they have nonetheless successfully guided bone regeneration in rat models in combination with RGD delivery [97] and in vitro with DNA encoding bone morphogenetic proteins [98]. They have also been used to transport whole cell populations (rat calvarial osteoblasts in a murine model [99], and osteoblasts with primary chondrocytes [100]) to take a direct part in new bone formation.

Alginate is also one of the most commonly used biomolecules in 3D bioprinting due to its compatibility with extrusion and inkjet printing processes [310], and its ability to maintain cell growth due to its similar properties to natural ECM [85,309]. As noted above, the ability of alginate hydrogels to bear weight and maintain their shape is limited, however, and so they are typically reinforced with natural and/or synthetic molecules such as fibrin or fibrinogen, nanocellulose, gelatin and nano-hydroxyapatite (n-HAP) to improve their structure [311].

Alginate has shown considerable potential as a biomaterial, particularly in wound healing, guided tissue engineering and drug delivery. While in hydrogel form, it exhibits very promising gelling capability, ease of modification and biocompatibility, the poor mechanical strength of other alginate forms limits their potential applications. Future research is likely to see the development of dressings with much higher bioactivities, as well as modified carrier vehicles for drug and bioactive molecules. Functionalisation with a wider range of molecules and engineering the molecules’ physical and chemical properties will be key to developing application-specific, tissue-interactive alginate biomaterials.

## 4. Conclusions and Future Perspectives

Natural polymers, both protein- and polysaccharide-based, display enormous potential in tissue engineering, drug delivery and diverse biomedical applications. Traditional materials previously designed to be inert in order to be “biologically safe” no longer meet the needs of modern medicine, where biomaterials are designed to make extensive biological and physical interactions with tissues and cells for improved in vivo performance. The ECM-like characteristics of natural polymers, their similarities to many human proteins and their natural versatility have led to the modification of well-established biomaterials, as well as the development of new materials, that have found novel applications in tissue engineering, as implantable structures and as vehicles for controlled drug release.

Though enormous progress has been made in the field of nature-based biomaterials in recent decades, room for improvement remains. Novel biomaterials which outperform current state-of-the-art offerings in terms of reduced side effects, lower purification or manufacturing costs and greater therapeutic efficacies will continue to be developed. Researchers continue to search for new natural sources of biomaterials which, together with innovative scaffold fabrication techniques, will lead to more effective biomaterials and new applications. The recent successes with natural materials and the broad array of materials currently available will inspire researchers to continue to look to nature for novel biomaterials, however, with fundamental biological and chemical understanding of such materials remaining central to the emergence of new, innovative biomaterials for new biomedical applications.

## Figures and Tables

**Table 1:** Summary of the different protein and polysaccharide-based biomaterials, their sources, main properties, structural forms used in biomedicine and biomedical applications.

Material	Source	Properties	Structures	Biomedical Applications
Collagen	Natural ECM or recombinant	Weakly immunogenic Cross-linked to increase strength, stability Cell binding	Scaffolds Sponges Hydrogels Films/membranes Bioinks	Tissue repair [34,35,36,37,38] Wound care [39,40,41] Drug delivery [42,43,44,45]
Gelatine	Bovine or porcine collagen	Biocompatible Biodegradable Cross-linked to increase strength, stability Cell binding	Microparticles	Drug delivery [46,47,48,49,50,51,52] Tissue regeneration [53]
Silk	Butterflies/moths, spiders or recombinant	High strength-to-density Insoluble in water Highly stable	Films Woven meshes	Wound dressings [22,54,55] Suturing [56,57] Device coatings [58,59]
Cellulose	Plants, bacteria	Biocompatible Combine stiffness and flexibility Tuneable properties	Nanofibres Gels Nanocrystals	Tissue engineering [60,61] Artificial blood vessels [62,63,64] Drug delivery [65,66,67] Wound repair [68,69,70,71,72,73,74,75,76,77]
Chitosan	Exoskeleton of crustaceans; plant cell envelopes	Rigid structure Insoluble in water Biodegradable Antimicrobial Versatile	Films Fibres Scaffolds Hydrogels Nanoparticles	Wound healing [78] Anti-microbial coatings [79,80] Drug delivery [81,82,83,84]
Alginate	Brown algae	Widely available Inexpensive Biodegradable Excellent gelating	Hydrogels Sponges Films Microparticles	Wound healing [85,86,87,88,89] Drug delivery [90,91,92,93,94,95] Tissue engineering [94,96,97,98,99,100]

## Data Availability

Not applicable.
